# An immunosuppressed microenvironment distinguishes lateral ventricle–contacting glioblastomas

**DOI:** 10.1172/jci.insight.160652

**Published:** 2023-06-22

**Authors:** Todd Bartkowiak, Sierra M. Lima, Madeline J. Hayes, Akshitkumar M. Mistry, Asa A. Brockman, Justine Sinnaeve, Nalin Leelatian, Caroline E. Roe, Bret C. Mobley, Silky Chotai, Kyle D. Weaver, Reid C. Thompson, Lola B. Chambless, Rebecca A. Ihrie, Jonathan M. Irish

**Affiliations:** 1 Department of Cell and Developmental Biology, Vanderbilt University, Nashville, Tennessee, USA.; 2 Department of Pathology, Microbiology and Immunology;; 3 Vanderbilt-Ingram Cancer Center; and; 4 Department of Neurosurgery, Vanderbilt University Medical Center, Nashville, Tennessee, USA.; 5 Vanderbilt Brain Institute, Vanderbilt University, Nashville, Tennessee, USA.

**Keywords:** Immunology, Oncology, Bioinformatics, Brain cancer, T cells

## Abstract

Radiographic contact of glioblastoma (GBM) tumors with the lateral ventricle and adjacent stem cell niche correlates with poor patient prognosis, but the cellular basis of this difference is unclear. Here, we reveal and functionally characterize distinct immune microenvironments that predominate in subtypes of GBM distinguished by proximity to the lateral ventricle. Mass cytometry analysis of isocitrate dehydrogenase wild-type human tumors identified elevated T cell checkpoint receptor expression and greater abundance of a specific CD32^+^CD44^+^HLA-DR^hi^ macrophage population in ventricle-contacting GBM. Multiple computational analysis approaches, phospho-specific cytometry, and focal resection of GBMs validated and extended these findings. Phospho-flow quantified cytokine-induced immune cell signaling in ventricle-contacting GBM, revealing differential signaling between GBM subtypes. Subregion analysis within a given tumor supported initial findings and revealed intratumor compartmentalization of T cell memory and exhaustion phenotypes within GBM subtypes. Collectively, these results characterize immunotherapeutically targetable features of macrophages and suppressed lymphocytes in GBMs defined by MRI-detectable lateral ventricle contact.

## Graphical abstract

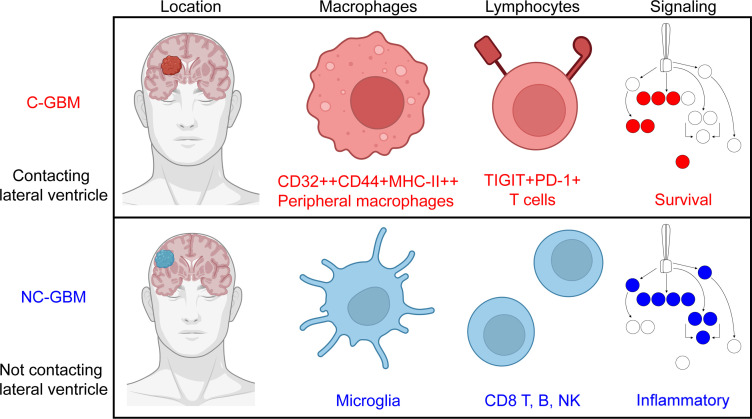

## Introduction

Glioblastoma (GBM) is the most common primary brain tumor, accounting for up to 60% of all brain tumors (1). Despite standard-of-care chemo/radiotherapy, median overall survival remains 15 months postdiagnosis (2). Tumor-treating fields or vaccines (DCVax-L) have improved survival to 20.0 and 19.3 months, respectively (3, 4); however, factors in the tumor microenvironment that contribute to improved survival have not been fully explored. While efforts have been made to phenotypically and molecularly characterize GBM tumors and illuminate the surrounding stromal elements influencing gliomagenesis, few cell-intrinsic factors beyond isocitrate dehydrogenase (*IDH*) mutation and O^6^-methylguanine-DNA methyltransferase (*MGMT*) promoter methylation have proven effective at stratifying patient outcome and guiding clinical care (5). Additional less invasive, risk-stratifying features are needed to make headway in understanding the complex cellular milieu in the tumor and advance therapeutics in the clinic.

The majority of GBM tumors present in the cerebrum, and although GBM may arise anywhere within the brain parenchyma, both adult and pediatric patients with primary high-grade gliomas that exhibit radiographic contact with the lateral ventricles (LVs) have worse prognosis (6, 7). This effect is independent of other predictive factors, such as patient age, performance status, or molecular characterization (8). Although regional tumor position stratifies prognosis, which factors proximal to the LV contribute to poor prognoses remain unclear.

Immune cells are an important component of the tumor lesion and play a critical role in controlling tumor growth in both solid and hematologic malignancies. The immune microenvironment within the brain, however, is distinct from peripheral tissue, and the antitumor potential of leukocytes in this discrete environment remains poorly characterized (9). Cytometric profiling has demonstrated regional differences in resident immune cell phenotypes under homeostatic conditions, particularly near the LV (10); however, how regional neuro-immunity is affected in the context of primary tumor lesions at the ventricle remains poorly understood. Here, we build on the concept that LV contact influences GBM prognosis and hypothesize that the immune microenvironment of tumors demonstrating radiographic contact with the LV (contacting GBM, C-GBM) will be distinct from those located distally from the ventricle (noncontacting GBM, NC-GBM).

In this study, we performed multidimensional single-cell mass cytometry on 32 primary human GBM tumors demonstrating radiographic contact with the LV (C-GBM) or located distally from the ventricle (NC-GBM) to comprehensively profile the immune infiltration in tumors within each region. Mass cytometry provides the advantage of resolving rare cell populations with a large dynamic range and the ability to measure cell surface receptors and phospho-signaling proteins. We developed 3 mass cytometry panels focused on interrogating immune abundance, checkpoint receptor expression, and protein phosphorylation in tumor-infiltrating lymphocytes, M1-like and M2-like monocyte-derived macrophages, and tissue-resident microglia, focusing on targetable immune receptors (e.g., programmed cell death 1 [PD-1] and T cell immunoreceptor with Ig and ITIM domains [TIGIT]) to test the hypothesis that the immune microenvironment of C-GBM tumors is more immunosuppressed. These markers were selected based on prior observations of myeloid-derived suppressor cells in cancer and definitional features of M2-like macrophages generated with cytokines (11).

Supervised and unsupervised machine learning approaches including clustering tools FlowSOM (12) and root mean square deviation (RMSD) (13), population identification via Marker Enrichment Modeling (MEM) (14), and patient stratification using Citrus (15) and Risk Assessment Population Identification (RAPID) (16) identified distinct immune subsets enriched in C-GBM and NC-GBM that correlated with patient outcome. Moreover, several targetable immune receptors were elevated in C-GBM tumors, suggesting that an immunosuppressive environment lies proximal to the LV. Lymphocytes and tissue-resident microglia were enriched in NC-GBM tumors and correlated with more favorable outcome, whereas antiinflammatory M2-like monocyte-derived macrophages (MDMs) bearing a distinct CD44^+^CD32^+^HLA-DR^+^ phenotype and exhausted PD-1^+^TIGIT^+^ T cells were enriched in C-GBM tumors.

Phospho-specific mass cytometry revealed broad T cell signaling defects and differential use of myeloid signaling networks employed by C-GBM and NC-GBM leukocytes in response to inflammatory cytokine stimulation. CD32^+^HLA-DR^+^ MDMs were highly responsive to cytokines compared with their CD32^–^HLA-DR^–^ counterparts. Further, STAT3 phosphorylation dominated signaling networks in C-GBM immune infiltrates compared with NC-GBM infiltrates, highlighting a potential STAT3-driven immunosuppressive mechanism in C-GBM tumors. This work highlights differing immune microenvironments within MRI-defined regional tumor classes, suggests distinct and targetable mechanisms of immune dysregulation in GBM tumors in relation to the LV, and emphasizes the potential for radiographic image–guided patient stratification methods to inform clinical care.

## Results

### Peripheral immune cells are abundant in GBMs.

**Figure 1:**
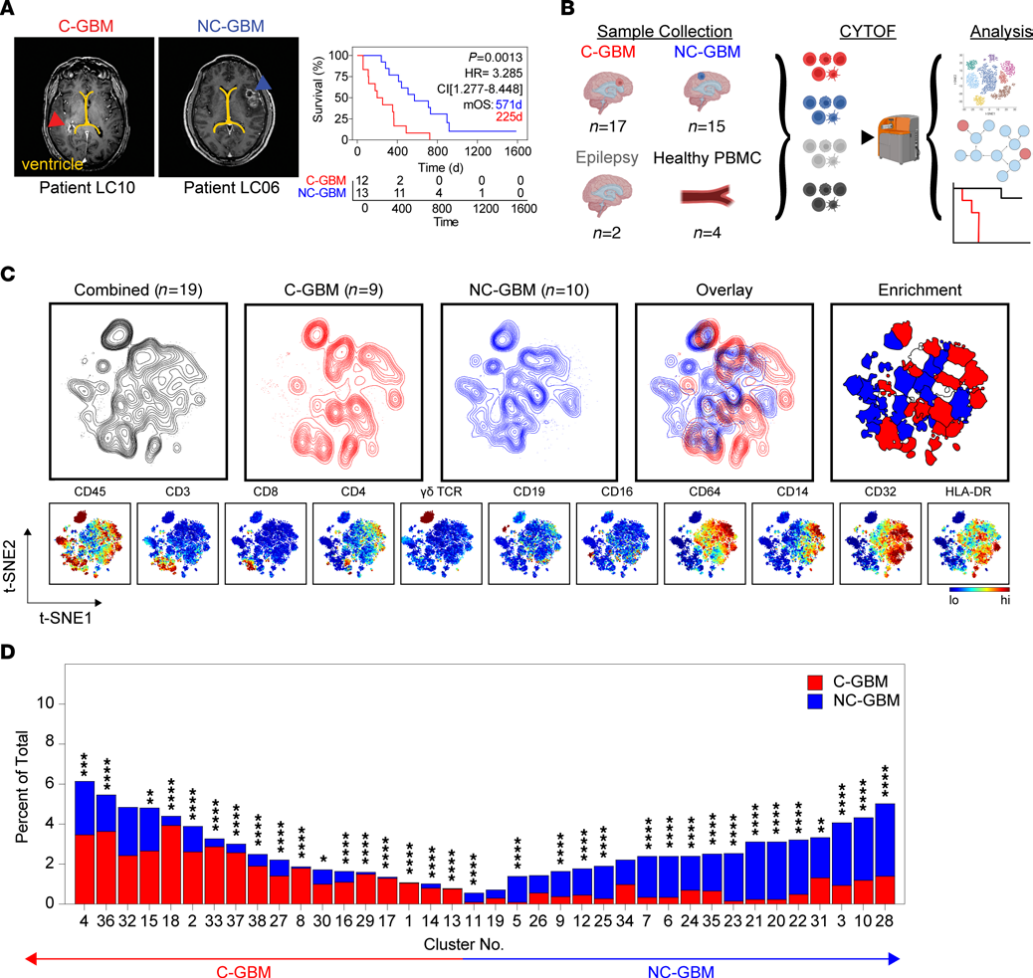
LV-contacting and -noncontacting GBMs are enriched in distinct immune subsets. (**A**) Representative MRI radiographs of GBM tumors with confirmed contact with either of the LVs (left, C-GBM) or lacking ventricular involvement (right, NC-GBM). Yellow line indicates the LV. Arrows indicate the tumor mass. Kaplan-Meier curve indicates the survival proportion in patients with C-GBM (*n* = 12) and NC-GBM (*n* = 13). (**B**) Schematic of experimental workflow. (**C**) Live CD45^+^ cells were combined from all patients (black contour, *n* = 19), C-GBM tumors only (red contour, *n* = 9), or NC-GBM tumors only (blue contour, *n* = 10). Overlaid t-SNE plots indicate areas of immune infiltration unique to patients with tumor subtype. Enrichment indicates which computationally gated immune populations were statistically enriched in C-GBM or NC-GBM. Heatmaps displayed for chosen markers indicate major immune subsets. (**D**) Bar graphs demonstrating the frequency of immune cells found within each computational cluster as a percentage of total CD45^+^ leukocytes. Statistical significance was calculated using a χ^2^ test. * = *P* < 0.05, ** = *P* < 0.01, *** = *P* < 0.001, **** = *P* < 0.0001.

To compare the immune microenvironment of GBMs contacting the LV to tumors distally oriented from the ventricle, we cytometrically profiled the cellular composition of 32 freshly resected GBM tissues from patients with radiographic contact with the LV (Figure 1, A and B). The median overall survival was 571 days for patients with NC-GBM and 225 days for C-GBM. *IDH1/2*-mutated tumors were excluded. Twenty patients (63%) were male, and 12 (37%) were female. Seventeen patients (10 male, 7 female) presented with C-GBM tumors and 15 patients (10 male, 5 female) with NC-GBM tumors. Ninety-four percent of patients (30/32) received steroids, 26/32 patients (81%) received temozolomide, and 30/32 (94%) received radiation. Twenty patients (63%) received subtotal surgical resection, and 12 (37%) received gross total resection (Supplemental Tables 1 and 2; supplemental material available online with this article; https://doi.org/10.1172/jci.insight.160652DS1). The median progression-free survival was 185 days (range: 22–1,539 days), and the median overall survival was 358.5 days (range: 57–1,588 days). Median survival in this cohort was within the 95% CI for studies of *IDH*-WT GBM. Aside from ventricle contact status, no single clinical factor was predictive of survival, as reported (2).

We first compared the tumor immune composition with that of healthy donor peripheral blood mononuclear cells (PBMCs) or epileptic brain tissue (Supplemental Figure 1A). T cell infiltrate in GBM, particularly CD4^+^ T cells, was similar to epileptic brain, accounting for 11% and 6% of the total leukocyte infiltrate, respectively. Other lymphocyte populations — Tregs, CD4^+^CD8^+^ T (DPT) cells, CD4^–^CD8^–^ T (DNT) cells, natural killer (NK) T cells, γδ T cells, B cells, and NK cells — each made up less than 5% of the leukocyte fraction in the tumor. CD45^lo^CD64^+^CD14^–^ microglia were the most abundant leukocyte population in the brain, accounting for 36% of leukocytes in GBM tumors. CD45^hi^CD64^+^CD14^+^ peripheral monocytes/MDMs were the next most abundant population, accounting for 32% of leukocytes in glioma, compared with 4% monocytes in PBMCs and 5% macrophages in epileptic brain, consistent with peripheral myeloid infiltration in GBMs (17).

As lymphocytic infiltration correlates with more favorable outcomes in peripheral solid tumors (18), we hypothesized that total leukocyte abundance in NC-GBM may drive favorable outcomes in this cohort. While the total leukocyte abundance in C-GBM and NC-GBM was higher than epileptic brain, no significant difference was found between the 2 patient cohorts, accounting for 23% (range: 0.47%–61.62%) and 20% (range: 0.38%–77.87%) of the tumor mass, respectively (Supplemental Figure 1B). The ratio of lymphoid cells (T, B, NK) and phagocytes (MDMs and microglia) was not significantly different between C-GBM and NC-GBM, suggesting that neither total leukocyte abundance nor bulk lymphocyte abundance fully accounted for differences in survival outcome between patients with C-GBM and NC-GBM. By overlaying leukocytes onto t-distributed stochastic neighbor embedding (t-SNE) axes, we identified distinct leukocyte populations in PBMCs, epileptic brain, and GBM tissue. The phenotypes of macrophages in C-GBM and NC-GBM tumors differed, occupying distinct islands within the t-SNE space (Supplemental Figure 1C), suggesting that phenotypic differences in immune infiltration correlated with ventricle contact status. For example, C-GBMs were infiltrated with CD45^hi^CD64^+^CD14^+^HLA-DR^hi^CCR4^hi^CXCR3^hi^CD69^+^CD56^hi^ macrophages compared with macrophages in NC-GBMs, or CD45^lo^CD64^+^CD44^lo^CD11b^lo^CD14^–^ microglia.

To further explore the extent of these cohort-level differences in immune abundance, we investigated the immune composition from 19 patients with GBM (9 C-GBM, 10 NC-GBM). Live CD45^+^ cells were equally sampled from each patient prior to plotting on common t-SNE axes using 33 measured dimensions (Figure 1C). Differences in immune composition were evident when comparing the distribution of C-GBM and NC-GBM samples across the common axes. FlowSOM clustering on the t-SNE axes defined 38 phenotypically distinct immune cell clusters across the patient cohort (Figure 1C and Supplemental Figure 2A). The majority of patients were well represented, with 2 to 17 patients contributing to each cluster (Supplemental Figure 2B). Of the 38 clusters, 17 were statistically enriched in leukocytes from patients with C-GBM, and 17 were enriched in leukocytes from patients with NC-GBM. Four clusters (clusters 19, 26, 32, 34) were not statistically enriched in either cohort (Figure 1D and Supplemental Figure 2, C and D). Taken together, these data highlight extensive differences in the immune composition of GBM tumors and suggest that while immune infiltration occurs in both C-GBM and NC-GBM, the relative composition of the immune fraction in ventricle-contacting tumors is distinct from that of ventricle-noncontacting tumors.

### Five immune cell subsets are differentially enriched in C-GBM and NC-GBM.

**Figure 2:**
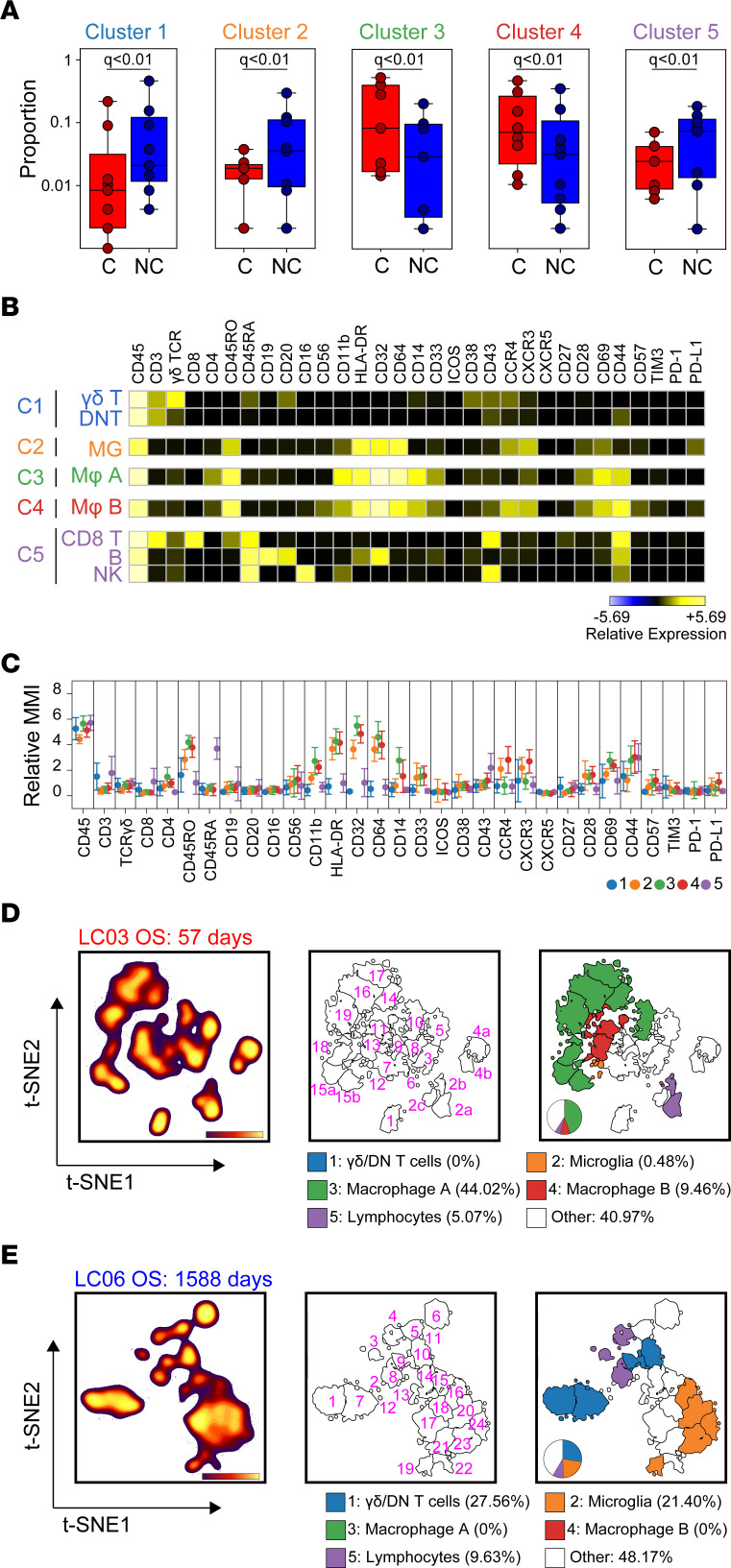
Differential enrichment of 5 immune phenotypes distinguish ventricle-contacting and -noncontacting GBM. (**A**) Citrus clustering of live CD45^+^ leukocytes in the tumor microenvironment of C-GBM and NC-GBM tumors revealed differential enrichment of 5 immune subsets. (**B**) Heatmaps of each phenotypic marker used to classify each immune subset reveal the expression levels of each immune receptor. (**C**) Quantification of the arcsinh-transformed expression level of each immune marker within each subset. MMI, median mass intensity. Representative t-SNE plot of all CD45^+^ leukocytes infiltrating a C-GBM tumor (**D**) or NC-GBM tumor (**E**). Cell density (left), FlowSOM clustering on the t-SNE axes (middle), and Citrus overlay and quantification (right) determined the relative frequency of each immune cell subset within each patient sample. In **A**, a regularized regression model in the Citrus analysis identified stratifying clusters (19 patients: 9 C-GBM, 10 NC-GBM). Predictive analysis of microarrays–stratified (PAM-stratified) immune clusters. An FDR < 1% (*q*) determined significance in all instances.

We next used machine learning to perform unbiased computational analysis of the immune microenvironment of GBM tumors and identify the most distinct immune subsets associated with C-GBM and NC-GBM tumors. Using the Citrus algorithm to compare leukocyte phenotypes and frequencies between our 2 cohorts (15), we identified 10 immune clusters differentially enriched in C-GBM and NC-GBM tumors. Five terminal clusters representing the most phenotypically distinct populations were selected for further study (Supplemental Figure 3A). Three Citrus clusters (clusters 1, 2, and 5) were enriched in NC-GBM, while 2 clusters (clusters 3 and 4) were enriched in C-GBM (Figure 2A). The identities of these clusters inferred from Citrus were confirmed via traditional biaxial gating and computational labeling using normalized, scaled MEM protein expression values (see Methods) (14) (Supplemental Figure 4, A–E). Cluster 1, NC-GBM, consisted of γδ T cells and CD3^+^CD4^–^CD8^–^ T (DNT) cells previously identified in GBM (13). Cluster 2 was a population of CD45^lo^CD64^+^CD14^-^HLA-DR^+^CD32^+^ resident microglial cells (Supplemental Figure 4B). Cluster 5, enriched in NC-GBM, consisted of lymphocytes: CD8^+^ T cells (28%), B cells (21%), NK cells (11%), and CD4^+^ T cells (7%) (Supplemental Figure 4E). In contrast, clusters 3 and 4, enriched in C-GBM, were characterized as CD45^hi^CD64^+^CD14^+^, consistent with peripheral MDMs (11) (Supplemental Figure 4, C and D). Expert-guided gating on patient samples excluded from Citrus analysis (*n* = 6) identified similar immune phenotypes consistent with computational findings (Supplemental Figure 3, B–D) and validated statistical enrichment of the immune populations identified by the Citrus algorithm (Supplemental Figure 3E).

Examining each cluster more deeply, we found γδ T cells in cluster 1 expressed CD45RA, CD38, CD43, and CCR4, while DNT cells expressed CD43 and CD44, consistent with an effector T cell phenotype (Figure 2, B and C). CD8^+^ T cells in cluster 5 expressed CD43, CD44, and CD45RA, indicative of activated CD45RA-expressing effector memory T (TEMRA) cells (19). B cells in NC-GBM tumors expressed low levels of HLA-DR and high levels of the inhibitory Fc receptor CD32 (FcγRII), suggesting impaired antigen presentation capacity. NK cells within cluster 5 were characterized as CD16^+^CD56^–^CD43^+^CD44^+^CD11b^lo^, a mature, cytotoxic NK cell phenotype. Finally, microglia enriched in NC-GBM (cluster 2) were characterized as CD45RO^lo^CD11b^lo^HLA-DR^+^CD32^+^CD64^+^CCR4^lo^CD69^lo^PD-L1^lo^, characteristic of activated microglia. In contrast, MDMs within C-GBM (clusters 3 and 4) expressed higher levels of CD45RO, CD11b, CD32, and CD44 than their microglial counterparts. The distinguishing characteristics of macrophage populations A (cluster 3) and B (cluster 4) included higher expression of CD14 in cluster 3 and higher expression of the chemokine receptors CXCR3 and CCR4 in cluster 4 (Figure 2, B and C), verifying that these populations represent 2 activated, phenotypically distinct myeloid subsets in the C-GBM microenvironment. We validated infiltration of these immune subsets within the tumor parenchyma using multiplex IHC (Supplemental Figure 5). In a cohort of patients with matched CyTOF and IHC data, we found equivalent frequencies of total T cells (CD3^+^), cytotoxic T cells (CD3^+^CD8^+^), helper T cells (CD3^+^CD4^+^), Tregs (CD3^+^CD4^+^Foxp3^+^), MDMs (CD68^+^ ionized calcium-binding adapter molecule 1–negative [IBA1^–^]), or microglia (CD68^+^IBA1^+^) identified by histochemistry or within single-cell suspensions, using either low-dimensional methods or multidimensional assessment.

Citrus events were then overlaid on patient-specific t-SNE maps (Supplemental Figure 6) to determine the source of subsampled Citrus clusters and compare the phenotypes and frequencies of immune cells with larger FlowSOM subsets (16). Immune phenotypes identified in the subsampled set of cells were not statistically different from the larger FlowSOM populations (*P* = 0.6844) (Supplemental Figure 6, A–D). Consistent with the abundance of leukocyte populations discussed above, the average number of phenotypically distinct FlowSOM clusters, an estimate of overall immunological diversity, was similar between C-GBM and NC-GBM tumors (23 vs. 22 FlowSOM clusters, respectively), as were the number and proportion of clusters represented by Citrus in the data set (Supplemental Figure 6E), suggesting that the degree of diversity within the immune infiltrate does not significantly contribute to outcomes associated with ventricle contact status.

The frequency and immune phenotype of individual patients’ clusters defined by MEM labels (Figure 2, D and E; Supplemental Figure 6F; and Supplemental Figure 7, A and B) reflected the patterns identified by Citrus. For example, patient LC03, who had a C-GBM tumor and the worst overall survival (57 days postresection), possessed abundant frequencies of macrophage population A (Citrus cluster 3, 44%; 8/23 clusters) and population B (Citrus cluster 4, 9.46%; 2/23 clusters). Five percent of leukocytes in this patient consisted of lymphocytes (CD8^+^ T cells [4%] and B cells [1%]), with less than 1% of the microglia and γδ/DNT populations (clusters 1 and 2) (Figure 2D and Supplemental Figure 7A). Conversely, patient LC06, who had an NC-GBM tumor and the greatest overall survival (1,588 days postresection) lacked macrophages in clusters 3 or 4 but had an abundance of microglia (21%), DNT cells (17.87%), γδ T cells (9.69%), NK cells (2.37%), CD8^+^ T cells (3.39%), and B cells (3.87%), collectively reflecting phenotypes found in clusters 1, 2, and 5 (Figure 2E and Supplemental Figure 7B). We next sought to compare the phenotypes of all individual FlowSOM immune clusters identified across all patients in order to identify common immune populations across our cohort. Comparison of all clusters (~23 clusters/patient) identified by FlowSOM (455 clusters from all patients in total) using RMSD identified populations enriched in C-GBM or NC-GBM tumors overlapping with Citrus phenotypes (Supplemental Figure 8A). RMSD analysis identified 17 common immune phenotypes across all patients. The majority of RMSD clusters were well represented across the cohort, with RMSD cluster 6 (CD8^+^ T cells) the most represented cluster and RMSD cluster 12 (microglia) the least represented (Supplemental Figure 8B). The abundance of each cluster within each patient sample (Supplemental Figure 8C), the statistical enrichment (Supplemental Figure 8D), and MEM labels for each immune phenotype (Supplemental Figure 8E) were consistent with Citrus findings.

Taken together, these data indicate that although individual patients possessed a range of immune cell phenotypes in their tumors, common immune signatures could be identified across the cohort. In particular, microglia and lymphocytes (γδ T cells, DNT cells, CD8^+^ T cells, NK cells, and B cells) were enriched in patients with NC-GBM. Conversely, patients with C-GBM possessed an immune microenvironment enriched in MDMs, revealing starkly contrasting immune microenvironments in C-GBM and NC-GBM tumors.

### Immune cell frequencies stratify patient outcome.

**Figure 3:**
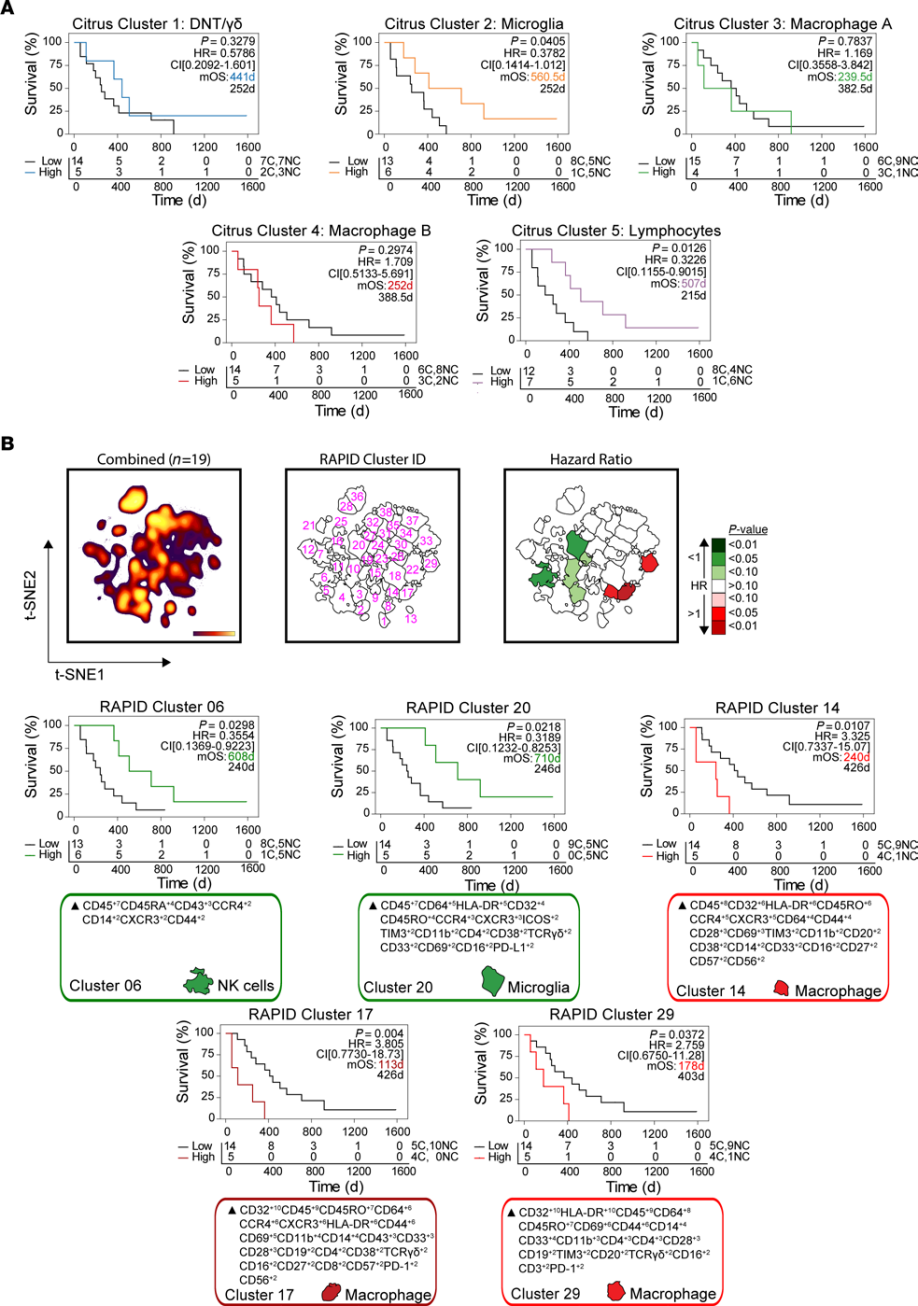
Immune subset frequencies correlate with patient outcome. (**A**) Kaplan-Meier curves indicating overall survival (OS) in GBM patients (*n* = 19) with high versus low frequencies of Citrus-identified immune populations. (**B**) Kaplan-Meier curves for OS in immune subsets stratifying patient outcome identified by RAPID analysis. T-SNE plots indicate the cell density (left), cluster number (middle), and *P* value of the HR associated with the frequency of each cluster in the entire cohort. Calculated MEM labels identified key features of stratifying immune subsets. *P* < 0.05 was considered significant.

We next hypothesized that the relative abundance of immune populations correlated with patient outcome, similar to observations in peripheral solid tumors (20). Using a Cox proportional hazards model, γδ T cells and DNT cells (Citrus cluster 1) and macrophages (Citrus clusters 3 and 4) did not significantly correlate with patient outcome (Figure 3A). Higher frequencies of microglia cluster 2 (>2%) correlated with more favorable overall survival outcomes (median 560.5 days vs. 252.0 days; *P* = 0.0405, HR = 0.3782, CI [0.1414–1.012]) as did lymphocyte cluster 5 (>6%) (median 507 days vs. 215 days; *P* = 0.0126, HR = 0.3226, CI [0.155–0.9015]). Importantly, 5/6 patients in the “microglia-high” and 6/7 patients in the “lymphocyte-high” group presented with NC tumors, consistent with our previous findings (see Figure 2). Outcomes associated with Citrus cluster frequency were not associated with patient sex (Supplemental Table 2) (21).

To corroborate these results, we used an orthogonal, unsupervised computational approach, RAPID, to directly reveal immune cluster abundances correlated with patient outcome (16). RAPID identified 8 populations that were either strongly (*P* < 0.05) or moderately (*P* < 0.1) correlated with patient outcome based on Kaplan-Meier survival estimation (Figure 3B). These phenotypes were stable through iterative runs of RAPID, including repeated subsampling to account for sampling bias (Supplemental Figure 9, A and B). Consistent with Citrus results, 1 statistically significant cluster (RAPID 06) represented a population of CD43^+^ NK cells whose abundance predicted favorable outcomes (*P* = 0.0298, HR = 0.3554, CI [0.1369–0.9223]). Five out of 6 patients with high frequencies of these NK cells presented with NC tumors, consistent with Citrus results. Greater frequencies (>1.32%) of RAPID cluster 20 (CD45^int/lo^CD64^+^HLA-DR^+^CD45RO^lo^ microglia) correlated with longer survival (710 days vs. 246 days; *P* = 0.0218, HR = 0.3189, CI [0.1232–0.8253]). All 5 patients with high abundance of this microglial subset presented with NC-GBM tumors. Conversely, 3 subsets of CD45^hi^CD64^+^CD32^+^HLA-DR^+^CD14^+^ macrophages correlated with poor prognosis (RAPID clusters 14, 17, and 29). RAPID cluster 17, an MDM population phenotypically similar to Citrus cluster 4 (macrophage population B), correlated with roughly 4-fold worse prognosis (median overall survival = 113 days vs. 426 days, *P* = 0.004, HR = 3.805, CI [0.7730–18.73]). Critically, patients with the highest frequencies of MDMs presented with C-GBM, providing further evidence that blood-derived macrophages correlate with ventricle tumor contact and worse prognosis. No association between RAPID-identified immune populations and patient sex was observed (Supplemental Table 2).

Collectively, these data suggest that infiltration by specific leukocytes correlates with survival in GBM and LV contact status. Orthogonal stratification approaches using RAPID and RMSD identified cells that aligned with subsets of microglial cells and lymphocytes identified by Citrus that correlated with more favorable outcomes, whereas increased infiltration of subsets of peripheral macrophages correlated with worse outcome. Thus, C-GBM and NC-GBM have distinct immune microenvironments and contrasting patient outcomes that align closely with the expected functional role of the immune cells present in each.

### Enriched checkpoint receptor expression in C-GBMs.

**Figure 4:**
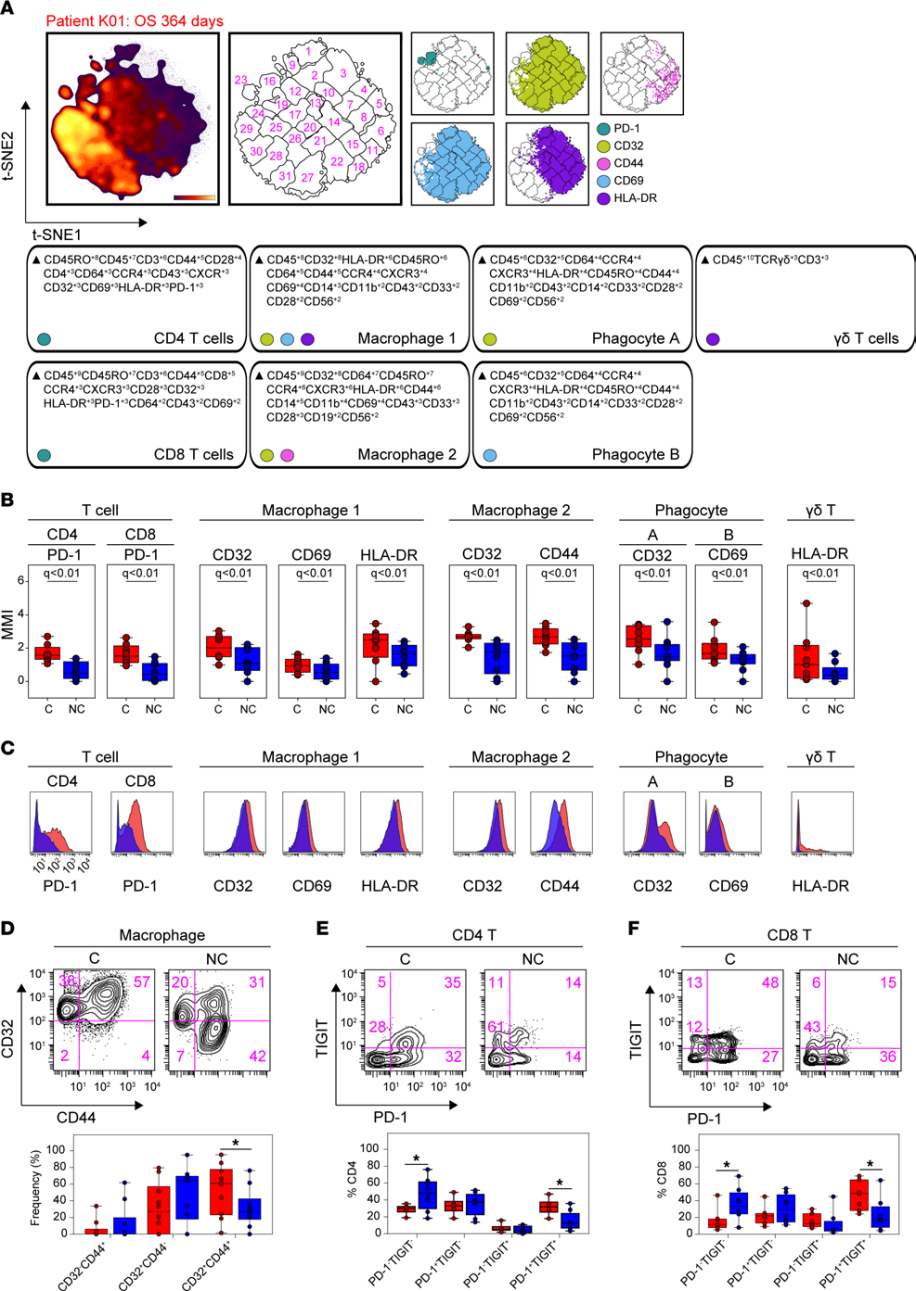
Immunosuppressive checkpoint receptors are enriched in ventricle-contacting GBMs. (**A**) Representative t-SNE plot (*n* = 19) indicating the density of all CD45^+^ leukocytes, FlowSOM clusters on the t-SNE axes, and overlaid immune populations with enriched expression of indicated immune markers. MEM labels indicate the cellular phenotype in which the indicated markers were differentially expressed. (**B**) Box-and-whisker plots indicating the arcsinh-transformed median expression values of indicated immune receptors within Citrus-identified populations (*n* = 19 patients). (**C**) Histograms of pooled patient Citrus clusters from C-GBM (red, *n* = 9) and NC-GBM patients (blue, *n* = 10). (**D**) Representative plots indicating the frequency of CD32^+^CD44^+^ macrophages identified by Citrus. (**E** and **F**) Representative plots demonstrating the frequency of TIGIT and PD-1 coexpression in CD4^+^ T cells (**E**) and CD8^+^ T cells (**F**) infiltrating GBM tumors. In **A**, a regularized regression model in the Citrus analysis identified stratifying clusters (*n* = 19 patients). PAM-stratified immune clusters. An FDR of 1% (*q*) determined significance. A 2-way ANOVA determined significance in **D**–**F** from *n* = 20 total patients. Bars indicate median ± IQR. * = *P* < 0.05.

We next sought to identify targetable immune receptors enriched within C-GBM or NC-GBM immune infiltrates. Regularized regression within Citrus identified elevated expression of 5 markers on 7 immune subsets in C-GBM tumors (Figure 4A and Supplemental Figure 10, A and B). Of note, the checkpoint receptor PD-1 was elevated on CD4^+^ and CD8^+^ T cells infiltrating C-GBM tumors, as were CD32, CD69, CD44, and HLA-DR on C-GBM–infiltrating peripheral MDMs. Moreover, CD32 and CD69 were elevated across multiple phagocytic populations infiltrating C-GBM. Last, HLA-DR was elevated on a population of γδ T cells infiltrating C-GBM tumors (Figure 4, B and C). Consistent with Citrus results here and in Figure 2, expert-guided biaxial gating verified an increased frequency of CD32^+^CD44^+^HLA-DR^+^ macrophages infiltrating C-GBM compared with NC-GBM (53% vs. 31%) (Figure 4D). Expert gating verified increased frequencies of PD-1^+^ CD4^+^, CD8^+^, and DNT cells within C-GBM tumors (Supplemental Figure 10, C–L, and Supplemental Tables 3 and 4). Critically, PD-1^+^ T cells infiltrating C-GBM coexpressed the inhibitory receptor TIGIT at a higher frequency in C-GBM (46% vs. 23%), whereas a higher frequency of PD-1^–^TIGIT^–^ T cells were found in NC-GBM (36% vs. 17%), suggesting that C-GBM T cells may be more phenotypically exhausted compared with their NC-GBM counterparts (Figure 4, E and F). Additionally, C-GBM tumors bore increased frequencies of T and NK cells (Supplemental Figure 10, C–F), and myeloid (Supplemental Figure 10, G–I) and B cells (Supplemental Figure 10J), with an activated phenotype compared with NC-GBM tumors. While CD45^–^ tumor stroma in C-GBM and NC-GBM expressed a variety of immune checkpoint receptors/ligands, we found no difference in the frequency of checkpoint-positive tumor cells in C-GBM and NC-GBM tumors (Supplemental Figure 10K). Interestingly, increased frequencies of T cells expressing several markers (CD27, CD32, CXCR3, CCR7) suggested that these cells in C-GBM tumors possessed an immunologic memory phenotype. Indeed, the frequency of CD45RO^+^CCR7^+^ central memory T (Tcm) cells was elevated in C-GBM tumors (Supplemental Figure 10L). Consistent with Citrus results (Figure 2), increased frequencies of CD45RO^–^CCR7^–^ TEMRA CD8^+^ T cells were found in NC-GBM tumors.

We next sought to identify which of the differentially enriched immune markers identified by Citrus may help stratify patient outcomes. To do so, we developed the median marker implementation of RAPID (mmRAPID) whereby the median intensity values for select markers in RAPID clusters were correlated with patient outcome (see Methods). Median expression values of 12 immune receptors across 46 immune clusters correlated with patient outcome (Supplemental Figure 11A). Consistent with enriched immune receptor expression in C-GBM tumors (see Figure 4) and immune abundance correlating with outcome (see Supplemental Figure 9), a 2-fold increase in PD-1 expression in CD4^+^ T cells (mmRAPID cluster 9) (Supplemental Figure 11B) and CD8^+^ T cells (mmRAPID cluster 25) (Supplemental Figure 11C) correlated with 2-fold worse survival (215 days vs. 441 days and 240 days vs. 570 days, respectively). Importantly, 6/9 patients with C-GBM tumors had PD-1^hi^CD4^+^ T cell infiltration and 8/9 patients PD-1^hi^CD8^+^ T cell infiltration, whereas 2/10 patients with NC-GBM tumors had PD-1^hi^CD4^+^ T cell infiltration and 3/10 NC-GBM patients PD-1^hi^CD8^+^ T cell infiltration. Further, mmRAPID identified a 2-fold increase in CD32 expression on CD8^+^ T cells (mmRAPID cluster 24), which correlated with a 2.6-fold decrease in patient survival (Supplemental Figure 11D). Consistent with Citrus identification of elevated expression of CD44 on C-GBM–infiltrative macrophages, mmRAPID found that elevated expression of CD44 on macrophages correlated with worse survival (Supplemental Figure 11E). Elevated expression of CD69 on CD4^+^ T cells (Supplemental Figure 11F) correlated with poor outcome and LV contact, but elevated CD69 expression on microglia was associated with more favorable outcomes and NC-GBM tumors (Supplemental Figure 11G). Moreover, mmRAPID identified elevated HLA-DR expression on CD4^+^ T cells, DNT cells, peripheral MDMs, and B cells as predictive of poor outcome and ventricle contact (Supplemental Figure 11, H–K). Surprisingly, PD-L1 expression, a feature of NC-GBM–enriched microglia (see Figure 2), correlated with improved outcomes and favored patients with NC-GBM (Supplemental Figure 11L). Consistent with increased frequencies of CD27^+^ T cells in C-GBM tumors, elevated CD27 expression on CD4^+^, DNT, and CD8^+^ T cells correlated with worse prognosis and favored patients with C-GBM tumors (Supplemental Figure 11, M–O). While CD28 expression on microglia correlated with more favorable outcomes and NC-GBM tumors (Supplemental Figure 11P), CD38, CCR4, TIM3, and CD57 were associated with worse outcomes (Supplemental Figure 11, Q–T).

Taken together, these data identify a statistical association between immunoreceptor expression, LV contact, and patient outcome using 2 contrasting machine learning tools. Elevated receptor expression tied to contact status and patient outcome identified by mmRAPID was consistent with Citrus identification of enriched immune populations in C-GBM and NC-GBM tumors. Coexpression of multiple predictive markers within the same immune subset suggests that key immune populations (e.g., PD-1^+^CD27^+^ T cells or HLA-DR^hi^CD44^+^ MDMs) may be critical drivers of immunity within the periventricular tumor microenvironment.

### Leukocyte populations associated with C-GBM were enriched proximal to the LV.

**Figure 5:**
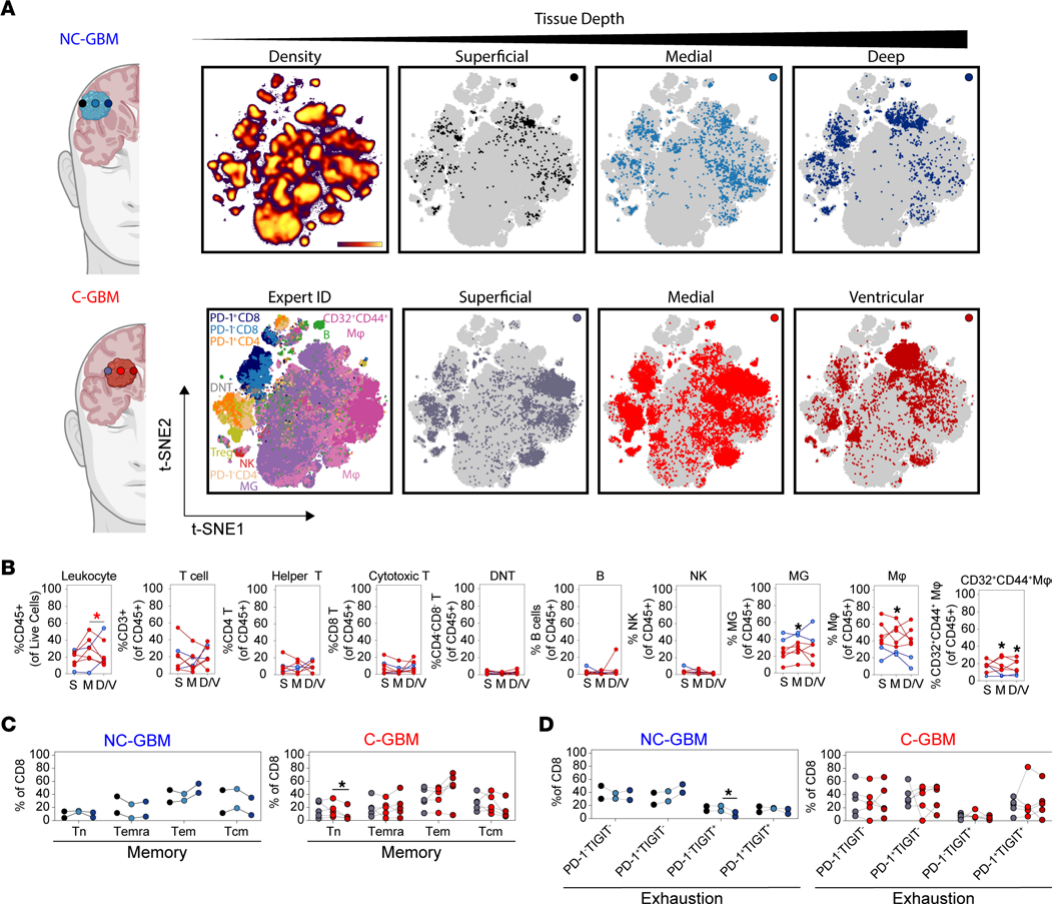
Enrichment of CD32^+^CD44^+^ macrophages proximal to the LV. (**A**) Representative t-SNE plots indicating the CD45^+^ leukocyte fraction infiltrating focal subregions biopsied from the bulk tumor mass of patients with NC-GBM (*n* = 2) or C-GBM (*n* = 5). Paired biopsies were collected from 1) superficial (black/gray), 2) medial (light blue/light red), or 3) deepest region available to safe surgical resection (dark blue/dark red). (**B**) The frequency of each indicated immune subset was calculated as a fraction of the total cell fraction in the biopsy (leukocytes) or as a fraction of the total leukocyte pool in each sample. S, superficial tumor tissue; M, medial tumor tissue; D/V, deep/ventricular tumor tissue. (**C**) Frequency of memory CD8^+^ T cell populations within subregions from NC-GBM (blue) or C-GBM (red). Tn, naive T; Temra, effector memory CD45RA^+^; Tem, effector memory; Tcm, central memory. (**D**) Frequency of exhausted CD8^+^ T cell populations within tumor subregions. Each line in **B**–**D** represents 1 paired patient sample. One-way ANOVA with Tukey’s multiple comparisons test calculated statistical significance. * = *P* < 0.05.

We used focal tissue collection to validate these results in 7 newly diagnosed GBM patients (5 C-GBM and 2 NC-GBM). Radiographically identified subregions were surgically resected from a) superficial tumor tissue proximal to the surgical incision site, b) the medial/core region, and c) the deepest tumor tissue antipodal to the surgical incision site, including ventricle proximal tissue in C-GBM tumors (Figure 5A). By overlaying each patient’s total leukocyte fraction onto common t-SNE axes, we identified immune populations infiltrating within each tumor subregion (Figure 5A). Total leukocyte abundance was highest within the core of C-GBM tumors and was significantly higher than in the ventricular region of C-GBM. No significant differences were identified in the overall abundances of T cell, B cell, NK cell, or phagocyte populations between subregions, suggesting that broad differences in immune infiltration between NC-GBM and C-GBM persist through the entire tumor mass. Microglia frequencies, however, were increased in NC-GBM tumors, in particular the core, compared with C-GBM. Reciprocally, MDM frequencies were increased in C-GBM tumors, especially the core, consistent with our previous results. While CD32^+^CD44^+^ macrophage frequencies were similar between C-GBM and NC-GBM in the most superficial regions, this subset was increased in the cores and deepest tissue of C-GBM compared with NC-GBM (Figure 5B). Given the high frequencies of both memory and exhausted T cell phenotypes in C-GBM tumors (see Figure 4 and Supplemental Figure 10), we investigated the location of these populations in the tissue. While memory T cell populations in NC-GBM were distributed throughout the tissue, TEMRA and effector memory T (Tem) cells appeared to be slightly enriched along the edges of the tumor compared with the core, and Tcm cells appeared reciprocally increased within the core compared with the edges. Within C-GBM tumors, naive T cells were reduced in samples proximal to the ventricle. Tem increased in frequency from the superficial to ventricular region, while Tcm cells decreased in proximity to the ventricle (Figure 5C). Similarly, there was an increase in PD-1^+^TIGIT^–^ and reduction in PD-1^–^TIGIT^+^ T cells with tissue depth in NC-GBM. PD-1^+^TIGIT^–^ T cells increased in frequency with proximity to the ventricle as did PD-1^+^TIGIT^+^ T cells (Figure 5D).

Taken together, these data suggest that not only does tumor proximity to the LV influence the types of immune cells infiltrating GBM tumors, but this contact also impacts the phenotype and potential function of these cells. Moreover, contact with the ventricle appears to influence the broader tumor microenvironment and does not merely influence immune infiltration into the most ventricle-facing tissue.

### Differential cytokine signaling capacity in the C-GBM tumor microenvironment.

**Figure 6:**
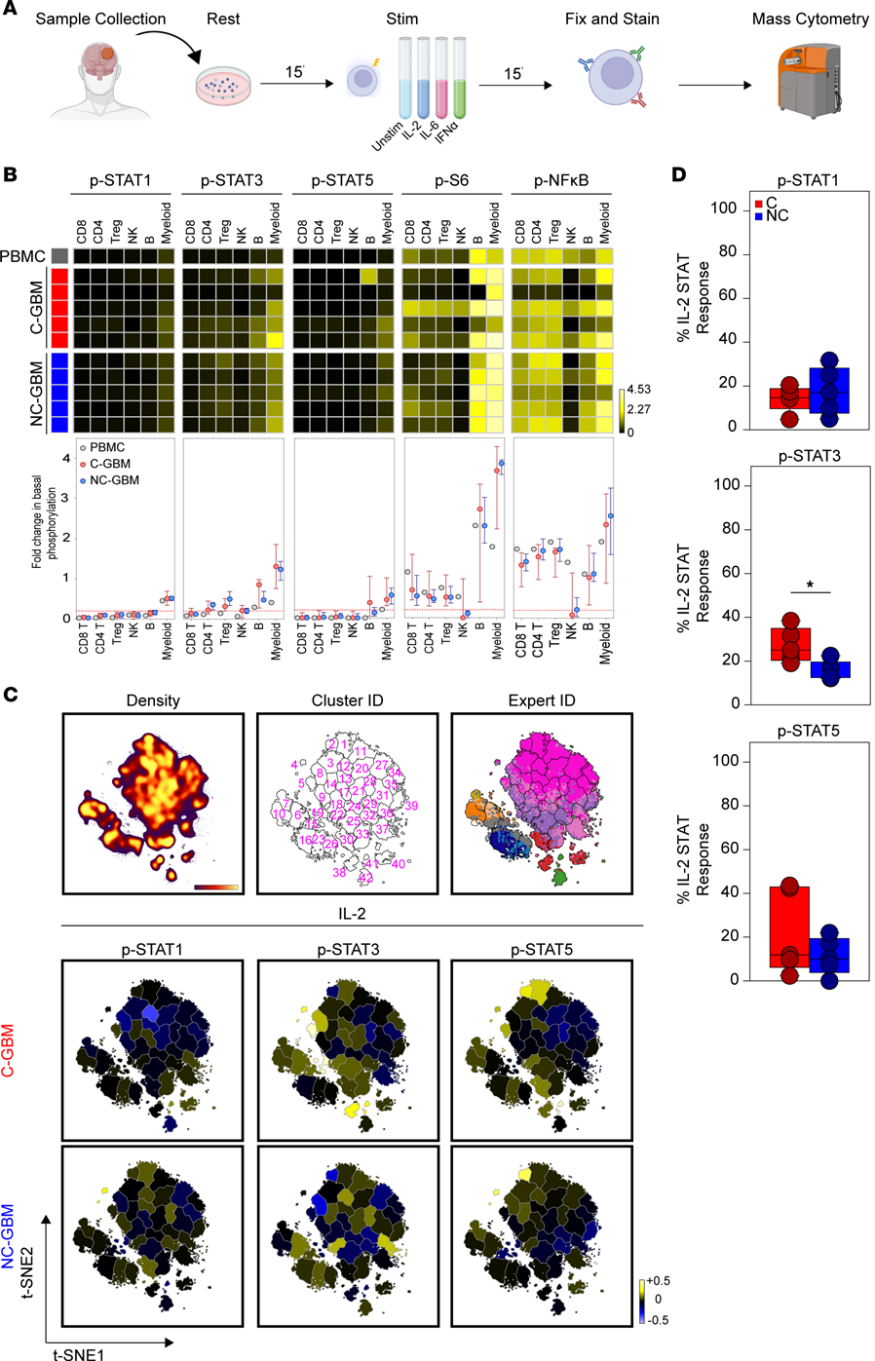
Immune cells infiltrating GBM tumors are functional and responsive to cytokine stimulation. (**A**) Schema of cytokine stimulation and phospho-protein readouts. (**B**) Heatmaps indicating the arcsinh fold-transformed median intensity values of each indicated phospho-protein within each manually gated immune subset in healthy donor PBMCs (gray, *n* = 1), C-GBM tumors (red, *n* = 5), or NC-GBM tumors (blue, *n* = 5). Graphs below the heatmaps indicate the median ± IQR for each indicated immune population and phospho-protein readout. (**C**) Representative t-SNE plot indicating the density of CD45^+^ leukocytes (left), enumerated FlowSOM clusters (middle), and overlay of expert-gated immune populations onto the clustered t-SNE axes (right) pooled from *n* = 10 patients. Representative heatmaps on the t-SNE axes indicate the cluster-specific median arcsinh fold-change of the indicated phospho-protein under IL-2 stimulation conditions compared with basal phosphorylation. (**D**) Box-and-whisker plots indicating the proportion of clusters in C-GBM or NC-GBM immune infiltrates surpassing the phospho-signaling threshold (>0.2 arcsinh fold-change) in response to IL-2 stimulation. Box-and-whisker plots indicate the median ± IQR.

Paradoxical coenrichment of activation markers (CD27, CD69, HLA-DR) and inhibitory receptors (PD-1) on the same lymphocyte subsets correlating with outcome suggested that these cells may retain functional capacity. To address the hypothesis that immune signaling differs between tumor types, we used mass cytometry to assess the basal levels of 12 phospho-proteins in immune infiltrates from 10 patients (5 C-GBM, 5 NC-GBM) or healthy PBMCs (Figure 6A and Supplemental Tables 1 and 5). Basal phosphorylated STAT1 (p-STAT1) occurred only in myeloid cells from PBMC or GBM samples; however, basal p-STAT3 was elevated in CD4^+^ T, Treg, B, and myeloid cells in GBM compared with healthy donor PBMCs, consistent with suppressive M2-like macrophages (reviewed in ref. 22). Further, p-STAT5 levels were increased in GBM-infiltrating myeloid cells. Ribosomal protein S6 (S6) and nuclear factor-κB (NF-κB) were phosphorylated in unstimulated T and B cells and were highly phosphorylated in myeloid cells. Contrastingly, NK cells exhibited impaired basal S6 and NF-κB phosphorylation, suggesting NK cell dysfunction, yet T, B, and myeloid populations appeared functionally competent (Figure 6B).

Given the coenrichment of immune activation and inhibitory receptors and correlations with worse survival seen in C-GBM, we next assessed the ability of leukocytes in C-GBM and NC-GBM tumors to respond to cytokine stimulation. Bulk tumor samples were stimulated ex vivo with cytokines with defined roles in antitumor immunity (interleukin-2 [IL-2], IL-6, and interferon-α [IFN-α]) (Figure 6A). FlowSOM clustering on t-SNE axes compared protein phosphorylation levels to basal signaling states in each of 42 immune subsets (Figure 6C). Quantification of the signaling responses for each of the 12 phospho-proteins within each cluster then identified signaling networks responsive to cytokine stimulation. Stimulation with IL-2 led to differential STAT phosphorylation within distinct immune subsets in C-GBM and NC-GBM tumors. For instance, STAT1 phosphorylation was refractory to IL-2 stimulation in C-GBM immune infiltrates, including cluster 12 (CD32^+^HLA-DR^hi^ MDMs [see Figure 4]). Conversely, cluster 2 (CD32^+^HLA-DR^+^ MDMs) phosphorylated STAT1 in response to IL-2 in NC-GBM tumors but not C-GBM tumors. Furthermore, IL-2 induced STAT3 phosphorylation in microglia (clusters 5 and 19) and NK cells (cluster 38) and STAT5 phosphorylation in CD32^+^HLA-DR^hi^ MDMs (clusters 2 and 4) and B cells (cluster 41) in C-GBM tumors. Immune infiltrates in NC-GBM tumors, however, exhibited a different pattern of IL-2–mediated STAT3 phosphorylation, as cluster 8 (microglia) and cluster 38 (NK cells) failed to phosphorylate STAT3. Similar to C-GBM, clusters 2 and 4 were responsive to IL-2–induced STAT5 phosphorylation, whereas cluster 41 (B cells) was refractory to STAT5 phosphorylation (Figure 6C and Supplemental Figure 12). The proportion of clusters responding to IL-2 through STAT signaling revealed that leukocytes in C-GBM tumors were significantly more responsive to IL-2 through STAT3 (25% of clusters) than NC-GBM infiltrates (12.5% of clusters) (Figure 6D). As for the signaling profile, IL-2 induced signaling cascades leading from membrane proximal signaling (LCK) to nuclear signaling (NF-κB) in several populations (e.g. cluster 2, 4, and 5) while other subsets (e.g., cluster 41) were reciprocally impacted by IL-2 stimulation through multiple networks (Supplemental Figure 12A).

Analogous to IL-2, IL-6 stimulation induced differential phospho-signaling in C-GBM and NC-GBM immune infiltrates (Supplemental Figure 13, A and B). STAT3 phosphorylation was induced in CD32^+^HLA-DR^+^ MDMs (clusters 2 and 21), microglia (cluster 5), DNT cells (cluster 15), and B cells (cluster 41) infiltrating C-GBM. However, these subsets failed to elicit IL-6 responses in NC-GBM (Supplemental Figure 13, A and B). Interestingly, IL-6 failed to induce p-STAT3 in cluster 2 macrophages in patients with NC-GBM, yet STAT5 was preferentially phosphorylated in this subset. In fact, IL-6 preferentially induced STAT3 phosphorylation across all C-GBM immune subsets, while STAT5 was favored in NC-GBM subsets (Supplemental Figure 13C), highlighting that inflammatory stimuli may have different immunomodulatory effects depending on tumor proximity to the LV.

Stimulation with IFN-α further distinguished immune responses in C-GBM and NC-GBM tumors (Supplemental Figure 14A). Interestingly, a STAT1/STAT3/ERK/p38/cAMP response element-binding protein (CREB) circuit was active in microglia infiltrating C-GBM (cluster 5) but not NC-GBM. In fact, IFN-α induced similar inflammatory circuits in CD32^+^HLA-DR^hi^ macrophages in either C-GBM or NC-GBM. For example, STAT1, -3, and -4 were induced within cluster 2 from both cohorts; however, STAT5 was preferentially induced in NC-GBM clusters 2 and 5 as was an AKT/ERK/p38/CREB circuit specifically in cluster 2 from patients with NC-GBM (Supplemental Figure 14B), suggesting an active inflammatory response to IFN-α in this subset. Similar to IL-2 and IL-6, IFN-α stimulation favored STAT3 phosphorylation over STAT1 in patients with C-GBM, as 19% of clusters demonstrated STAT3 responses in C-GBM, whereas only 10% of clusters demonstrated STAT1 phosphorylation, further supporting a role for STAT3 in immune regulation in C-GBM tumors.

Together, these data indicate that not only do immune cells differentially infiltrate C-GBM and NC-GBM, but also the inflammatory milieu within C-GBM may rewire immune signaling networks, altering immune responsiveness to external stimuli. STAT3 phosphorylation drove much of the cytokine responsiveness in C-GBM tumors regardless of the cytokine stimulation or expected canonical signaling pathways, consistent with our hypothesis that C-GBM tumors possess a distinct STAT3-driven immunosuppressive microenvironment.

## Discussion

Significant improvements in molecular and histologic characterization have increased our understanding of neurologic tumors, including GBM. Unfortunately, improved tumor classification has not translated into clinical therapeutics that meaningfully influence patient outcomes. Work over the past decade has focused on tumor-specific characterization and targeting, and only recently has an impetus been placed on understanding stromal factors that drive gliomagenesis and therapeutic resistance. The immune microenvironment, in particular, constitutes a critical part of tumor lesions and plays a crucial role in regulating tumorigenesis (23, 24). While immune-targeted drugs have generated antitumor immunity toward peripheral solid tumors and can mediate complete tumor regression, the same efficacy has not been demonstrated in neurologic tumors (ClinicalTrials.gov NCT02017717, NCT02667587) (25, 26). Immunotherapeutic strategies have demonstrated some capacity to elicit antitumor immunity (4, 27), however, highlighting a need for novel, straightforward approaches, such as MRI-guided regional tumor position, to identify appropriate immune targets and patient cohorts to optimize therapeutic benefit.

Here we describe the immune microenvironment in GBMs based on regional tumor position, identifying immunomodulatory mechanisms in GBM tumors presenting with radiographic contact with the walls of the LV. Complementary mass cytometry, IHC, and machine learning approaches provided supervised and unsupervised approaches identifying phenotypic and functional immune profiles associated with ventricle contact status and patient outcome. Namely, lymphocytes and tissue-resident microglia were more abundant in NC-GBM tumors, whereas C-GBM tumors were enriched in antiinflammatory CD32^+^CD44^+^HLA-DR^hi^ M2-like MDMs and exhausted PD-1^+^TIGIT^+^ T cells. These observations suggest that C-GBM tumors, and potentially the periventricular space itself, are highly immunosuppressive environments.

Several lymphocyte subsets were enriched in NC-GBM tumors, including γδ T cells and CD8^–^CD4^–^ DNT cells, as well as CD8^+^ T cells, NK cells, and B cells, which correlated with improved patient survival. γδ T cells and DNT cells generate neuro-inflammatory responses during brain pathology and are associated with antitumor cytotoxicity in GBM (13, 28, 29). CD45RO^–^CCR7^–^ CD8^+^ TEMRA cells were also enriched in NC-GBM tumors. Although TEMRA cells are terminally differentiated, they possess increased cytotoxicity and tumor-killing capacity (19, 30), suggesting that TEMRA cells may contribute to tumor control in NC-GBM tumors. Moreover, NK cells infiltrating NC-GBM tumors lacked CD56, associated with potent toxicity, whereas CD56^hi^ NK cells infiltrating C-GBM tumors suggest a regulatory capacity (reviewed in refs. 31, 32). Infiltration of lymphocyte subsets with cytotoxic potential in NC-GBM suggests a more effective antitumor immune response occurs in this microenvironment, contributing to longer patient survival. Furthermore, B cells were enriched in NC-GBM. B cell–enriched tertiary lymphoid structures (TLSs) in tumors correlate with improved patient survival and responsiveness to immunotherapy (33–35); however, the precise role of B cells and their ability to support TLSs in NC-GBM remains to be seen.

**Figure 7:**
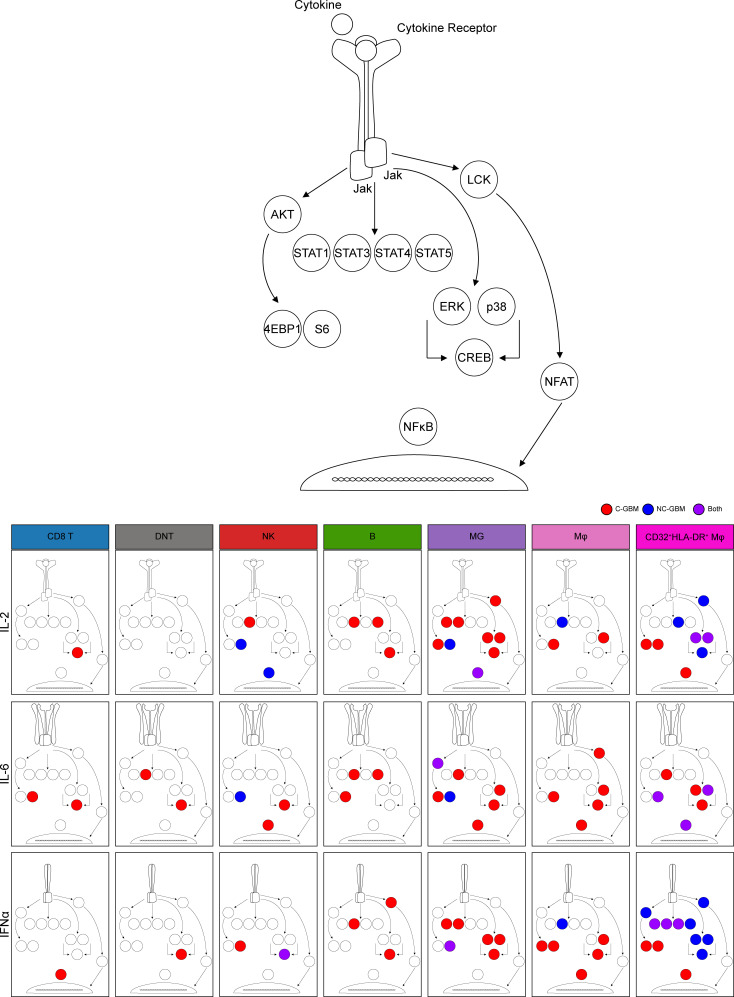
Model of cell signaling networks in GBM immune infiltrates. Graphical representation of immune cell signaling networks. For each cytokine stimulation condition implemented (rows) and each cell population of interest (columns), an aggregate signaling diagram was generated. Signaling nodes in red indicate active signaling responses to the indicated cytokine stimuli in C-GBM tumors, and blue nodes indicate active signaling responses in NC-GBM. Purple nodes indicate protein phosphorylation in response to stimuli in both patient cohorts.

In contrast to NC-GBM tumors, lymphoid infiltration into C-GBM was reduced, and lymphocytes in C-GBM tumors bore a hyperactivated/exhausted phenotype characterized by increased expression of CD32, CD69, HLA-DR, CD27, and PD-1 on T cells that correlated with worse prognosis. An increased frequency of PD-1^+^ T cells infiltrating C-GBM coexpressed the TIGIT checkpoint receptor. While the precise mechanism of TIGIT-mediated immune suppression is unclear, TIGIT serves a compensatory role in PD-1–mediated inhibition, eliciting resistance to PD-1–targeted checkpoint blockade therapy (36). Moreover, TIGIT expression is associated with elevated T cell activation, immune exhaustion, and responsiveness to checkpoint blockade (37–39). Functionally, T cells in C-GBM and NC-GBM lacked Granzyme B and Ki-67, suggesting a limited effector function and proliferative capacity consistent with immune exhaustion. In contrast to TEMRA cell infiltration in NC-GBM, CD45RO^+^CCR7^+^ Tcm cells predominated in C-GBM tumors, largely at the ventricle-distal edge. In fact, several T cell markers correlating with patient outcome (CD32, CD69, HLA-DR, CD27) are associated with T cell memory. Whether TEMRA and Tcm cells differentially infiltrate NC-GBM and C-GBM tumors, or are polarized by microenvironmental factors, and functional consequences upon arrival remain to be determined. Increased frequencies of multiple CCR7^+^ lymphocyte populations in C-GBM tumors suggest that a CCR7/CCL19/CCL21 axis may recruit lymphocytes into the periventricular niche. Importantly, T cells in C-GBM and NC-GBM tumors exhibited basal phosphorylation of S6 and NF-κB, indicating incomplete exhaustion and retention of some degree of functionality. While lymphocytes in both C-GBM and NC-GBM showed impaired responses to inflammatory cytokines (Figure 7) (arguing for exhaustion), lymphocytes in C-GBM tumors demonstrated moderate responses to inflammatory cytokines, particularly through CREB, suggesting that these lymphocytes may retain tonic signaling, preventing apoptosis, and may respond to immune-targeted therapies. It remains to be seen what effects other cytokines and soluble factors may have on T cell signaling, recruitment, and survival in C-GBM and NC-GBM.

Tumor contact with the LV also influenced phagocytic populations in the tumor microenvironment. CD45^lo^CD11b^lo/–^HLA-DR^lo^CD14^–^ microglia were enriched in NC-GBM tumors and corresponded to favorable outcomes; however, no distinct phenotype emerged to illuminate their functional capacity. Microglia in NC-GBM tumors demonstrated limited responsiveness to inflammatory cytokines, with p-S6 remaining an active signaling component (Figure 7). Microglia contribute to tissue homeostasis through phagocytosis of cellular debris and tissue pruning (reviewed in ref. 40). The phagocytic and antigen presentation capabilities of NC-GBM microglia are unknown but may support infiltrating lymphocytes in mediating tumor control. In contrast with NC-GBM tumors, 2 populations of peripheral CD45^hi^CD11b^+^CD14^+^ MDMs distinguished by expression of CD32, CD44, HLA-DR, CD69, and chemokine receptors CXCR3 and CCR4 were enriched in C-GBM tumors. Along with elevated frequencies of CCR7^+^ lymphocytes, these findings support a role for chemotactic factors in recruiting leukocyte populations into the niche.

C-GBM–infiltrating MDMs expressed CD163 and CD209 (DC-SIGN), indicative of an M2-like antiinflammatory phenotype (41). MDMs exhibited high basal levels of p-STAT3, consistent with M2-like polarization. Interestingly, CD32^+^HLA-DR^+^ macrophages showed greater responses to cytokine stimulation than their CD32^–^HLA-DR^–^ counterparts (Figure 7). Upon stimulation, these MDMs utilized different signaling networks in C-GBM and NC-GBM tumors. CD32^+^HLA-DR^+^ macrophages signaled through a STAT3/4/5–ERK–p38–CREB axis in NC-GBM tumors in response to IL-2 stimulation. While this network was active in C-GBM MDMs, eukaryotic translation initiation factor 4E-binding protein 1 (4E-BP1), S6, and NF-κB were also phosphorylated, suggesting some degree of differential macrophage signaling based on ventricle contact. Similarly, C-GBM MDMs were more responsive to IL-6 stimulation, particularly through STAT3, in C-GBM compared with NC-GBM. CD32^+^HLA-DR^+^ MDMs were highly responsive to IFN-α stimulation. While all STAT proteins assessed were phosphorylated following IFN-α stimulation in C-GBM and NC-GBM MDMs, NC-GBM MDMs favored an inflammatory ERK/p38/CREB signaling axis, while C-GBM MDMs favored 4E-BP1, S6, and NF-κB signaling, further supporting a role for the tumor environment in mediating differential myeloid signaling responses. Recent reports have demonstrated conflicting roles for IFN-α signaling in mediating pro- or antitumor responses depending on the chronicity of IFN exposure (see ref. 42). Our results support a hypothesis that long-term IFN exposure in the ventricular space may negatively influence antitumor immunity, whereas acute inflammatory IFN signaling along with increased lymphocyte infiltrate support antitumor immunity and prolong overall survival in patients with NC-GBM tumors. Importantly, STAT3 phosphorylation took place in C-GBM immune infiltrates regardless of stimulation condition. This points to STAT3 as a critical, targetable driver of antitumor immunity in the ventricular space and suggests the stoichiometry of STAT3 may enforce a regulatory immune signaling axis; however, the ability of STATs to form heterodimeric complexes in response to inflammatory cues in brain tumors, and the resulting functional consequences, are poorly understood.

We assessed signaling responses to 3 inflammatory cytokines that impact antitumor immunity to peripheral solid tumors; however, a complex milieu of cytokines, soluble mediators, and unique parenchymal factors within the brain may further influence antitumor immunity in the brain. Identifying the cellular source (tumor, stem cell, ependyma, choroid plexus, or immune) and the dynamic interplay between these cell subsets within the microenvironment in mediating antitumor immunity will be critical to advance our understanding of neuro-oncology and develop novel therapeutics.

This work highlights potential immunotherapeutic targeting strategies for patients with GBM based on MRI-guided tumor proximity to the LV. Future studies will be needed to determine whether patients with NC-GBM or C-GBM tumors may be more amenable to drug combinations targeting either lymphocyte or MDM populations, respectively, depending on the tumor microenvironment and regional position. Immuno-oncology agents currently approved or in clinical trials may afford the most immediate benefit, particularly for patients with C-GBM tumors, including agents targeting PD-1 (nivolumab, pembrolizumab), TIGIT (tiragolumab), CD27 (varlilumab), or STAT3 (WP-1066). It remains to be seen, however, which immunotherapeutic combinations will improve outcomes in patients with GBM.

The tumor immune microenvironment is heavily influenced by tumor tissue of origin, particularly in the brain (e.g., brain metastases possess distinct immune microenvironments dependent on tissue of origin, ref. 43). Here, we demonstrate that regional position of primary brain lesions, visualized as MRI-guided contact with the LV, influenced antitumor immunity in the brain and will be germane to clinical decision-making, particularly in patient selection and therapeutic options available to patients with C-GBM versus NC-GBM.

## Methods

### Human specimens.

Freshly resected GBM tissues were collected from the Department of Neurosurgery at Vanderbilt University Medical Center between 2014 and 2018 (16, 44). Noncancerous brain tissue was collected from temporal lobectomy for epilepsy from the Veterans Affairs Medical Center affiliated with Vanderbilt University. GBMs confirmed by PCR as *IDH* mutant were excluded. All patients were adults 40–80 years old at the time of tumor resection. Extent of resection was classified as gross total resection (GTR) or subtotal resection independently by a neurosurgeon and a neuroradiologist. GTR was defined as no significant residual tumor enhancement upon gadolinium-enhanced MRI of the brain 24 hours postsurgery. Tumor contact with the LV was confirmed upon inspection of MRI and verified by a neurosurgeon and neuroradiologist. The subventricular zone spans the frontal, temporal, occipital, and atrial compartments of the LVs. Therefore, invasion, contact, or containment of any region of the LV was considered “ventricle-contacting.” All patients were considered for postoperative chemotherapy and radiation. Methylation of the MGMT promoter was determined by pyrosequencing (Cancer Genetics Inc). Patient follow-up extended to October 2019, noting time to patient’s death. All deaths were deemed related to tumor progression. Median overall survival of the patient cohort was 358.5 days. A complete list of clinical characteristics can be found in Supplemental Tables 1 and 2.

PBMCs were collected from healthy volunteers with written informed consent under IRB protocol 131311 in accordance with the Declaration of Helsinki. Samples were deidentified prior to processing. No other information was obtained from healthy individuals.

### Tissue collection and processing.

Fresh tumor tissue was obtained directly from the operating room at Vanderbilt University Medical Center within 1 hour of resection. Tissues were processed and dissected into single-cell suspensions as reported (44). Samples were resuspended in DMEM-F12 with glutamine (Glutamax, Thermo Fisher Scientific), 1 M HEPES (Gibco), hormone cocktail (30% glucose [Thermo Fisher Scientific], 7.5% sodium bicarbonate [MilliporeSigma], apotransferrin, insulin, Putrescine solution, 200 μM progesterone, 3 mM sodium selenite [MilliporeSigma]), and gentamicin (Thermo Fisher Scientific). Tissues were minced to a diameter of 1 mm before enzymatic digestion for 1 hour with collagenase IV (1 μg/μL, MilliporeSigma) and DNase I (0.25 μg/μL, MilliporeSigma) at 37°C and 5% CO_2_ with steady shaking. Suspensions were washed and triturated before filtration through 70 μm and 40 μm filters (Corning). Cell pellets were resuspended in ACK lysis buffer to remove red blood cells (Invitrogen) and washed and resuspended in neural stem cell media with BSA (MilliporeSigma), heparin, recombinant human FGF (25 μg/mL; Stem Cell Technology), and recombinant human EGF (10 μg/mL; Stem Cell Technology) in 10% DMSO before cryopreservation (1 × 10^7^ cells/mL) in liquid nitrogen.

Healthy donor blood was collected by venipuncture into heparinized tubes (Becton Dickinson; 100 mL/donor). Whole blood was diluted 1:4 with PBS before overlay onto a Ficoll-Paque Plus density gradient (GE Life Sciences, now Cytiva). Blood was centrifuged at 400*g* for 30 minutes at room temperature sans brake. Buffy coats were isolated, washed with PBS, and centrifuged at 500*g* for 10 minutes at room temperature. Pellets were resuspended in ACK lysis buffer for 5 minutes, washed, and cryopreserved at 1 × 10^7^ cells/mL in liquid nitrogen in 10% DMSO/FBS.

### Metal isotope–tagged antibodies.

All antibodies used for mass cytometry analysis are listed in Supplemental Tables 3–5. Antibodies preconjugated to metal isotopes were purchased from Standard BioTools or commercial suppliers in purified form and conjugated in-house using the Maxpar X8 chelating polymer kit (Standard BioTools) according to the manufacturer’s instructions.

### Cell preparation and mass cytometry acquisition.

Cryopreserved samples were rapidly thawed in a 37°C water bath and resuspended in RPMI supplemented with 10% FBS and 50 U/mL of penicillin-streptomycin (HyClone, Thermo Fisher Scientific). Cell suspensions were processed and stained as described (44, 45). Cells were washed with serum-free RPMI and stained with 1 μM ^103^Rh Cell-ID Intercalator (Standard BioTools) for 5 minutes at room temperature before quenching with complete RPMI and washing with PBS/1% BSA. Cells were resuspended in PBS/BSA and added to an antibody cocktail of cell surface–staining antibodies and incubated at room temperature for 30 minutes. Samples were washed in 1% PBS/BSA before fixation in 1.6% paraformaldehyde (PFA) for 10 minutes at room temperature. Cells were washed in PBS and fixed in ice-cold methanol with gentle vortexing before storage at –20°C. On the day of data collection, samples were washed in PBS/BSA and resuspended in an antibody cocktail of intracellular stains for 30 minutes. Iridium Cell-ID Intercalator (125 nM) was added and incubated at room temperature for 30 minutes. Cells were washed and resuspended in ultrapure deionized water, mixed with 10% EQ Four Element Calibration Beads (Standard BioTools), and filtered through a 40 μm FACS filter tube before data collection on a Helios CyTOF 3.0 (Standard BioTools). Quality control and tuning processes were performed following the guidelines for the daily instrument operation. Data were collected as FCS files.

### Cytokine stimulation and phospho-specific cytometry.

Phospho-specific mass cytometry was performed as described (45). Cryopreserved samples were thawed in a water bath. Cell pellets were resuspended in RPMI (10% FBS, penicillin-streptomycin) and rested at 37°C for 15 minutes. Cell suspensions were washed in PBS and stained in 1 μM ^103^Rh Cell-ID Intercalator in PBS for 5 minutes. Cells were washed in PBS/BSA and aliquoted equally into cytokine stimulation solutions: PBS, recombinant human IL-2 (20 ng/mL), recombinant human IL-6 (20 ng/mL), recombinant human IFN-α (20 ng/mL), or hydrogen peroxide (10 μM). Stimulation conditions proceeded for 15 minutes before immediate fixation in 1.6% PFA to halt phospho-protein dissociation. Samples were washed in PBS/BSA, stained with a cocktail of cell surface antibodies, and fixed in ice-cold methanol. On the day of collection, samples were stained with a cocktail of intracellular phospho-specific antibodies before iridium intercalation, resuspension in EQ calibration beads, and sample collection.

### Data preprocessing.

Raw mass cytometry files were normalized using the MATLAB bead normalization tool before upload to the Cytobank platform. Before automated high-dimensional data analysis, mass cytometry data were transformed with a cofactor of 5 using an inverse hyperbolic sine (arcsinh) function. Cell doublets were excluded using Gaussian parameters (center, offset, width, residual) as reported (45). Intact cells were gated using DNA content (^191^Ir and ^193^Ir). Dead cells were excluded based on rhodium intercalation. Immune subsets were then manually gated using biaxial gating.

### Dimensionality reduction and automated clustering.

T-SNE analysis was performed on each individual patient sample in Cytobank. All live CD45^+^ cells were included for each patient’s t-SNE analysis (range: 1,322–335,303 events), including all immune markers in the antibody panel to generate t-SNE maps: perplexity = 30, theta = 0.5, and iterations = 10,000. Automated clustering of immune cell subsets was then performed for each patient using the FlowSOM tool in Cytobank using the t-SNE1 and t-SNE2 channels, hierarchical consensus clustering, cluster number = 196, and 10 iterations (12). Repeated FlowSOM analyses using different iterations identified the optimal number of metaclusters (*n* = 5–50) that minimized the variance of each immune marker as described (16). For patient-to-patient comparisons where indicated, an equal number of live CD45^+^ events were downsampled from each patient file, then concatenated, and the number of FlowSOM clusters was optimized across all patients.

### Citrus clustering.

To identify immune populations enriched in abundance in either C-GBM or NC-GBM, we used the Citrus algorithm in Cytobank (15, 46). Live CD45^+^ cells were equally downsampled from 19/32 patient files (478 events/patient) (Supplemental Table 1). Patient files included in the analysis were grouped by contact status (9 contacting, 10 noncontacting). All immune markers in the Immune Phenotyping Panel (Supplemental Table 3) were used to cluster. A nearest shrunken centroid predictive analysis of microarrays (PAMR) model predicted enriched cell abundance within each group. The minimum cluster size was set to 5%, with 5 cross-validation folds, and FDR = 1%. To determine enriched immune marker expression, the Citrus algorithm was rerun investigating median marker expression. Sixteen immune markers delineating lymphocyte and myeloid cell subsets clustered events, while the arcsinh expression values of 17 markers were explored (Supplemental Table 3). The maximum number of events per file was sampled using PAMR analysis, 5% cluster size, 5 cross-validation folds, FDR = 1%. The most terminal clusters of differential abundance between patient cohorts were exported for further analysis. Biaxial gating determined each cluster phenotype. To determine from which immune populations each downsampled Citrus cluster was sampled, t-SNE analysis was performed on the total number of live CD45^+^ cells from each patient and each patient’s individual Citrus clusters. Cells from Citrus clusters were assigned to FlowSOM metaclusters based on position in the viSNE map and phenotypic similarity. Cellular abundance with an identified Citrus phenotype and expression of identified markers were validated independently in the entire cohort.

### MEM.

The phenotypes of automatically clustered immune populations generated in Citrus and FlowSOM were identified using MEM (14). FlowSOM or Citrus clusters were exported from Cytobank into R, where MEM labels were generated using all immune markers included in the phenotyping (Supplemental Table 3) or the checkpoint panel (Supplemental Table 4). As a modification of the original MEM script, we compared marker expression within each cluster to a reference point wherein the magnitude of the median expression value of the null set was defined as 0, and the IQR was defined as the median IQR for all features in the MEM analysis (47). MEM values were then scaled from 0 (no expression) to 10 (high expression) relative to the reference point. Populations were hierarchically clustered using the hclust package in R based on median marker expression, MEM value, or IQR. MEM labels for each population were confirmed by biaxial gating.

### RMSD.

To compare intra- and interpatient phenotypic similarities between automatically clustered immune populations, MEM values were generated for each cluster. MEM labels were compared using the RMSD calculation in the “MEM_RMSD” function included in the MEM package in R (https://github.com/cytolab/mem). The “MEM_RMSD” function calculates the square root of the mean squared distance between every MEM value in common for a given pair of cell subsets. These values were transformed and expressed as a percentage of the maximum RMSD in the analysis. Heatmaps of each hierarchically clustered population based on RMSD score and a matrix of RMSD values were exported from R. MEM labels were generated by concatenating all clusters within a branch of the RMSD hierarchal clustering tree, and an average MEM value for each marker was generated.

### Analysis of phospho-signaling.

Analysis of immune cell phospho-signaling under different cytokine stimulation conditions was performed in Cytobank. Baseline phospho-signaling was compared in biaxially gated populations. Changes in protein phosphorylation under stimulation conditions were normalized to basal signaling in unstimulated FlowSOM clusters. An equal number of CD45^+^ events (3,523 cells/patient) was downsampled from each patient’s stimulation condition file before t-SNE analysis. All immune phenotypic markers were used to generate the t-SNE plot, and phospho-proteins were excluded. FlowSOM clustering was performed on the t-SNE axes, and the number of FlowSOM clusters was optimized to minimize variance. The median phospho-protein value from each stimulation condition was then compared with the median value from unstimulated cells using the median arcsinh transformation. A ≥ 0.2 or ≤ -0.2 fold-change in arcsinh-transformed median phospho-protein expression over baseline was considered a response.

### Multiplex IHC.

Cyclic IHC was performed as reported (48). Formalin-fixed, paraffin-embedded tissue was sequentially stained with a panel of validated antibodies (CD3, CD4, CD8, FOXP3, PD-1, CD68, IBA1, PD-L1) with colorimetric detection using 3-amino-9-ethylcarbazole via the Translational Pathology Shared Resource at Vanderbilt University Medical Center. Between rounds of staining, images were digitally acquired using a Leica SCN400 slide scanner. The Cyclic Analysis of Single-Cell Subsets and Tissue Territories image analysis pipeline registered sequential images and identified immune populations within the tissue (48).

### Survival analysis.

Correlations of immune subset abundance with overall survival were performed using RAPID in R (16). T-SNE analyses were performed by downsampling live CD45^+^ events from each patient file and optimizing the number of FlowSOM clusters. The frequency of cells from each patient within each cluster stratified patients into high or low cluster abundance based on the interquartile distribution of the subset across the entire cohort. A univariate Cox regression model then estimated the HR of death and determined statistical significance using the “survival” package in R. Overall survival was defined as time from surgical resection to death. Survival time was censored if, at last follow-up, the patient was known to be alive and had not had radiographic tumor progression. Differences in the survival curves were compared using a Cox univariate regression model, reporting an HR between the survival curves. Statistical significance was set at 0.05 for all statistical analyses. RAPID analysis was performed on 10 t-SNE analyses resampled from each patient. Clusters from each independent analysis that met an HR threshold >1 or <–1 and *P* < 0.1 were isolated, and RMSD was performed to determine cluster stability with similar phenotypes identified by RAPID.

mmRAPID correlated immune receptor expression with patient outcome. Dimensionality reduction was performed using t-SNE on 16 markers used in the Citrus median marker analysis (Supplemental Table 3). FlowSOM clustering on the t-SNE axes minimized the variance in marker expression across all clusters. The arcsinh-transformed median marker expression of 17 markers of interest stratified patients into high and low expression based on the interquartile distribution of the arcsinh-transformed values across the entire cohort for each cluster. A univariate Cox regression model estimated the HR of death. Marker expression was validated by biaxial gating and generation of MEM labels for high and low groups.

### Statistics.

Statistical analysis was performed in Cytobank as a part of advanced analyses, in R version 3.6.1, or in GraphPad Prism version 8.4.3 where indicated. All analyses were graphed in the GraphPad Suite. Outlier analysis was performed before all statistical analyses using the ROUT method (*Q* = 1%). Statistical analysis of 2 groups was performed using a 2-sided Student’s *t* test with Welch’s correction. Analysis of 3 or more groups was performed using a 1-way or 2-way ANOVA with a Tukey’s or Sidak’s correction for multiple hypothesis testing, respectively. Immune subset enrichment was statistically determined using a χ^2^ or Fisher’s exact test. Statistical correlations of immune subset abundance were performed using a 2-tailed Pearson’s correlation. For all statistical tests unless otherwise indicated, *P* values of less than 0.05 were considered significant. Graphs show median ± IQR unless otherwise indicated.

### Study approval.

All samples were collected consecutively with patient written informed consent in compliance with the Vanderbilt IRBs (030372, 131870, 181970) and in accordance with the Declaration of Helsinki. Samples were deidentified prior to tissue processing.

### Data availability.

Data sets analyzed in this manuscript manuscript are available online at FlowRepository (Repository ID: FR-FCM-Z5ZC, FR-FCM-Z5ZD, FR-FCM-Z5ZE, FR-FCM-Z5RH) (49). Transparent analysis scripts for data sets in this manuscript (first shown in Figure 3 and Supplemental Figure 11) will be publicly available on the CytoLab Github page (https://github.com/cytolab/GBM-IMM01; commit ID ca1c781) with open source code and commented Rmarkdown analysis walkthroughs.

## Author contributions

TB, RAI, and JMI designed the study. TB, MJH, JS, NL, and CER collected data. TB, SML, and AAB developed data analysis scripts. TB, SML, RAI, and JMI performed mass cytometry data analysis and interpretation. AMM and SC scored MRI images for tumor contact with the LV and provided patients’ clinical characteristics. BCM confirmed tissue pathology. LBC, RCT, and KDW provided freshly resected tissue specimens. TB, RAI, and JMI wrote the manuscript. RAI and JMI provided financial support. TB, SML, MJH, AMM, AAB, JS, NL, CER, BCM, SC, KDW, RCT, LBC, RAI, and JMI contributed to reviewing and editing the manuscript.

## Supplementary Material

Supplemental data

Supplemental table 1

Supplemental table 2

Supplemental table 3

Supplemental table 4

Supplemental table 5

## Version 1 05/16/2023

In-Press Preview

## Version 2 06/22/2023

Electronic publication
